# Childhood leptospirosis in an industrialized country: Population-based study in Okinawa, Japan

**DOI:** 10.1371/journal.pntd.0006294

**Published:** 2018-03-08

**Authors:** Kouki Tomari, Takao Toyokawa, Takuto Takahashi, Tetsuya Kakita, Sho Okano, Hisako Kyan, Naoya Tonegawa, Teppei Okawa, Takashi Matsuoka, Tsutomu Matsumora

**Affiliations:** 1 Department of General Pediatrics, Okinawa Prefectural Nanbu Medical Center & Children’s Medical Center, Okinawa, Japan; 2 Department of Infectious Disease, Okinawa Prefectural Nanbu Medical Center & Children’s Medical Center, Okinawa, Japan; 3 Department of Pediatrics, SUNY Downstate Medical Center, Brooklyn, NY, United States of America; 4 Medical Microbiology and Zoology Section, Okinawa Prefectural Institute of Health and Environment, Okinawa, Japan; University of Texas Health Science Center at Houston McGovern Medical School, UNITED STATES

## Abstract

Leptospirosis is considered underdiagnosed because of its nonspecific presentation and lack of proper understanding of its epidemiology. Early diagnosis and treatment are crucial. However, few data are available on confirmed leptospirosis cases in children in industrialized countries. We therefore aimed to describe epidemiologic and clinical characteristics of laboratory-confirmed childhood leptospirosis in Okinawa, Japan. We reviewed the national surveillance data of pediatric leptospirosis in Okinawa, Japan from January 2003 through December 2015. The database included all of laboratory-confirmed leptospirosis diagnosed at the only central laboratory for leptospirosis in the region. There were 44 children (0–20 years of age) with laboratory-confirmed leptospirosis. Of these, 90% were male, 91% were 10–20 years of age, and 96% of cases occurred in August and September. The number of laboratory-confirmed patients ranged from 0 to 11 per year (mean: 3.3 per year), and the estimated annual rate was 1.0 per 100,000 pediatric populations. In all cases, the presumed infection route was recreational exposure to river water. Commonly observed manifestations include fever (95%), myalgia (52%), and conjunctival suffusion (52%). Childhood leptospirosis in Okinawa, Japan occurred predominantly in teenage boys after freshwater exposure in summer, and most patients had characteristic conjunctival suffusion. Cohort studies would be helpful to better understand more detailed clinical manifestations in association with prognosis.

## Author summary

Leptospirosis is a bacterial disease that affects humans and animals. It is caused by Leptospira. Leptospira is a spiral-shaped Gram-negative spirochete with internal flagella, which enters the host through mucosa and broken skin. Leptospirosis is a worldwide zoonosis, but it is considered underdiagnosed because of its nonspecific presentation and lack of proper understanding of its epidemiology, especially in children. In human, it can cause a wide range of symptoms in acute phase include fever, chills, headache, muscle aches, vomiting, and diarrhea. Some of these symptoms can be observed in other febrile diseases such as typhoid fever, rickettsiosis, Kawasaki disease, and adenovirus infections. Although Leptospirosis can be deadly in severe, untreated cases, few data are available on leptospirosis in children, industrialized countries, and confirmed cases. This study conducted a population-based study to describe epidemiologic and clinical characteristics of childhood leptospirosis in a leptospirosis-endemic area in an industrialized country, Okinawa, Japan. This new knowledge may contribute to a better overall understanding of the Leptospirosis in children.

## Introduction

Leptospirosis is a neglected zoonotic disease with global distribution that is caused by infection of spirochetes from the genus *Leptospira*. Although leptospirosis usually presents as mild, self-limiting symptoms, severe cases with high mortality and morbidity might occur [1]. Therefore, early diagnosis and treatment are crucial to improve clinical outcomes [2]. However, its nonspecific presentation and lack of proper understanding of its epidemiology pose diagnostic challenges in non–leptospirosis-endemic areas despite the availability of confirmatory testing.

The epidemiology of leptospirosis is mainly reported from developing countries in the tropics. Although it is now recognized as a disease of global distribution, it is considered underreported in industrialized countries [3]. In addition, limited knowledge is available in pediatric patients with leptospirosis. Acquisition of *Leptospira* in human commonly occurs through contact with contaminated water or infected animals. Therefore, its prevalence might vary according to lifestyle factors or sanitary conditions. Another gap in leptospirosis research includes studies on confirmed cases in children. Reports from leptospirosis-endemic countries mainly diagnosed cases on the basis of clinical findings, which are known to be nonspecific. In addition, there is a limited number of large case series consisting of laboratory-confirmed cases, which mainly comprised adult patients [4–6]. Clinical investigation on laboratory-confirmed pediatric patients would aid in the understanding of the presentation of childhood leptospirosis.

Here, we conducted a population-based study to describe epidemiologic and clinical characteristics of childhood leptospirosis in a leptospirosis-endemic area in an industrialized country, Okinawa, Japan.

## Methods

### Setting

We retrospectively evaluated all leptospirosis cases reported in Okinawa Prefecture, Japan, which is the only subtropical and tropical zone in Japan. Okinawa is located in the southwestern part of the country and consists of 363 islands in the East China Sea and the Philippine Sea. Among them, there are 49 inhabited islands with a total population and a total child population (≤20 years of age) of approximately 1,400,000 and 330,000 persons, respectively.

### Data collection

We retrospectively reviewed pediatric patients (0–20 years of age) using the Japanese national surveillance data of leptospirosis from Okinawa from January 2003 through December 2015. Although older teenagers are physiologically like adults, we chose the age-range of 0–20 years to identify the pediatric patients because of admitting practices to hospitals in Japan. In Japan, hospitalized patients in the 0–20 year age group are most often seen by the Pediatrics service, while the Adult Internal Medicine service sees the older patients. The national surveillance data include date of onset, age, sex, diagnostic method, serovar, signs, symptoms, laboratory results, estimated incubation periods, and presumed exposure locations and activities (e.g., recreation or labor in fresh water). Leptospirosis is a nationally notifiable disease under the Japanese law, “Act on the Prevention of Infectious Diseases and Medical Care for Patients with Infectious Diseases.” Physicians are instructed to send blood, urine, and cerebrospinal specimens for microbiological testing for *Leptospira* during the acute phase (i.e., within a few days after the onset of symptoms) and the convalescent phase (i.e., 10–14 days later) when they examine patients with suspected leptospirosis. Okinawa Prefectural Institute of Health and Environment is the central laboratory for Leptospirosis in Okinawa and the institute grasp total number of laboratory-confirmed cases.

### Laboratory analysis and diagnostic definition

The diagnosis was made according to the definitive diagnosis criteria of leptospirosis by the National Institute of Infectious Disease, Japan: either positive serologic tests, isolation of *Leptospira* from a normally sterile site, or *Leptospira* DNA detected by polymerase chain reaction (PCR). Serologic testing was performed by the microagglutination test (MAT), and *Leptospira* was isolated mainly by using Korthof medium [7]. Diagnostic criteria for positive MAT were a four-fold or higher rise in titer between the acute and the convalescent phase. All laboratory analyses were conducted by Okinawa Prefectural Institute of Health and Environment, Okinawa, Japan.

### Data analysis

We calculated the mean annual rate using the mean child population during the study period. These figures were compared with previously reported adult data collected through the same surveillance program [8]. The estimated annual rate was 0.86 per 100,000 population in this adult population (i.e., >20 years of age; approximately 1,070,000 adults) during the study period. The population data were obtained from data of the Basic Resident Registration from Okinawa Prefectural Government [9].

### Ethics

This study was conducted by using the national surveillance data and information from Okinawa Prefectural Institute of Health and Environment and National Institute of Infectious Diseases in Japan according to a Japanese law. Therefore, written informed consent from individual patients was deemed unnecessary. In addition, our research met the Japanese government guidelines for conducting research, “Ethical Guidelines for Medical and Health Research Involving Human Subjects,” which applies to 1) research carried out pursuant to the provisions of laws and ordinances, and 2) research utilizing only specimens and information widely utilized in research and generally available, and information which has already been anonymized [10]. Therefore, Institutional Review Board approval was deemed unnecessary.

## Results

### Patient characteristics

**Fig 1:**
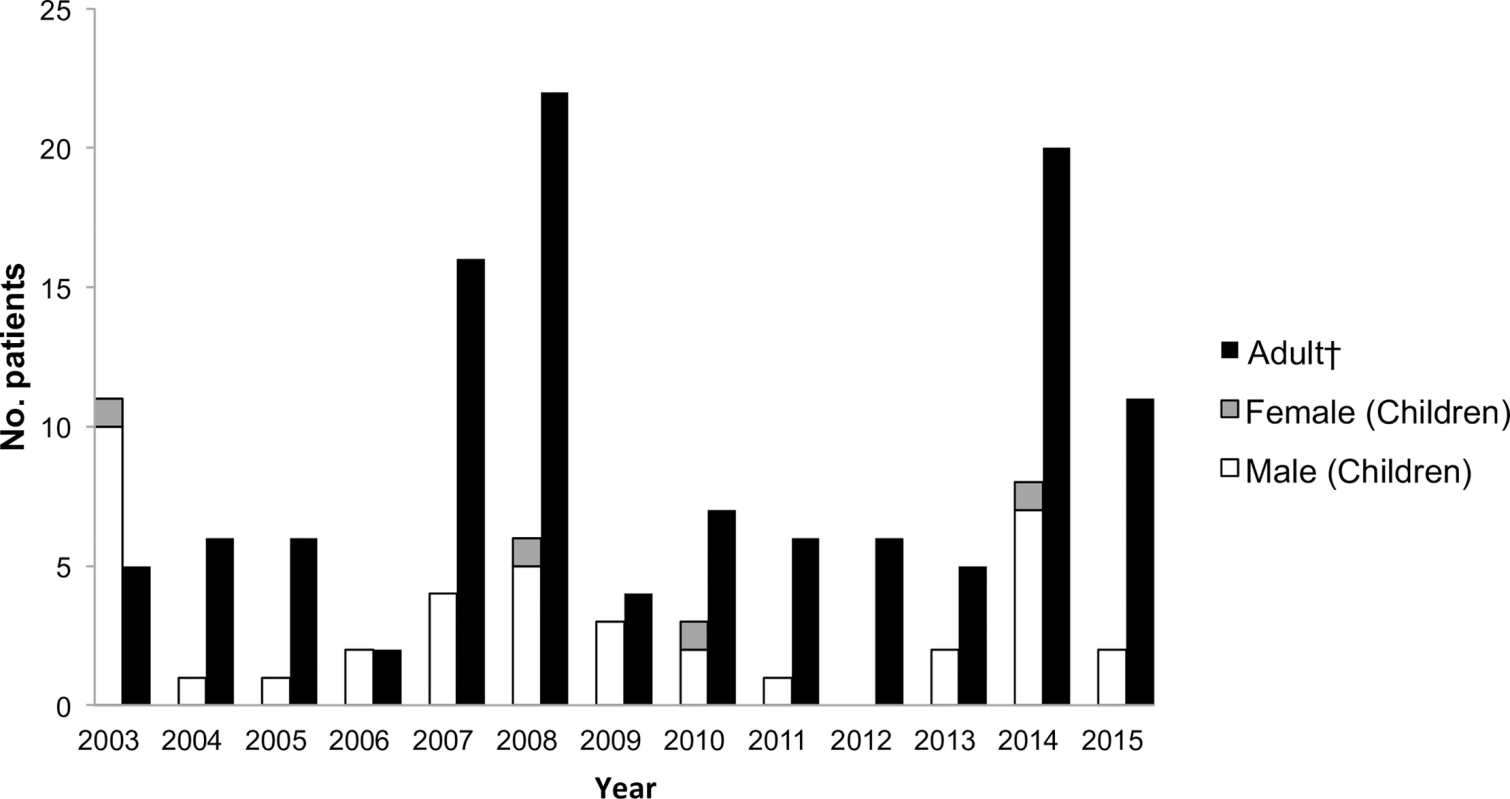
Annual number of cases. Adult leptospirosis cases reported in Okinawa [5] are shown for comparison.

Among the 155 patients with confirmed leptospirosis during the study period, 44 pediatric patients were included. Of these, 40 patients (90%) were male. The median age was 14 years (range: 5–20 years), and the number of patients ≤10 years of age was 4 (9%) (Table 1). The number of laboratory-confirmed patients ranged from 0 to 11 per year (mean: 3.3 per year), and the estimated annual rate was 1.0 per 100,000 population according to a pediatric population of approximately 330,000 children (Fig 1). The presumed infection route was predominately recreational or occupational activity in river water (e.g., swimming in a river) (41/44, 93%) (Table 1). All but two cases (96%) occurred in August and September; these two cases occurred during October and November (Table 1). The mean duration from the onset of symptoms to hospital arrival was 4.4 days. Presumed infection locations were limited to two areas, northern parts of Okinawa Island (31/44) and Yaeyama region (10/44); the location for three cases was not identified.

**Table 1:** Patient characteristics, no. (%).

	Participants
	n = 44
Male sex	40 (91)
Age in years, median (range)	14 (5–20)
0–10 years old	4 (9)
11–20 years old	40 (91)
Exposure	
Freshwater	41 (93)
Unknown	3 (7)
Season of onset	
August	11 (25)
September	31 (70)
October	1 (2)
November	1 (2)

### Signs and symptoms

Commonly observed manifestations included fever (95%), myalgia (52%), conjunctival suffusion (52%), and gastroenteritis-like symptoms (34%). Features of Weil’s disease, a severe form of leptospirosis, such as liver dysfunction, renal dysfunction, and jaundice occurred in 27%, 25%, and 13% of the patients, respectively (Table 2).

**Table 2:** Patient characteristics (n = 44), no. (%).

Clinical features		Serogroups of Leptospira*	
Fever	42 (96)	Hebdomadis	28 (64)
Myalgia	23 (52)	Autumnalis	8 (18)
Conjunctival suffusion	23 (52)	Pyrogenes	3 (7)
Gastroenteritis-like symptoms	15 (34)	Grippotyphosa	2 (5)
Liver dysfunction	12 (27)	Australis	1 (2)
Renal dysfunction	11 (25)	Cross-reaction	1 (2)
Arthralgia	10 (23)	Untested	1 (2)
Jaundice	6 (14)		
Headache	6 (14)		
Meningitis	4 (9)		
Lymphadenopathy	2 (5)		
Altered mental status	2 (5)		
Rash	1 (2)		

### Leptospirosis diagnostic tests

Among the 44 confirmed cases of leptospirosis, 18 cases were diagnosed only by serologic tests, 4 cases by isolation in culture, and 1 case by PCR. Eight cases were diagnosed by serologic tests and isolation, 1 case by serologic tests and PCR, 8 cases by isolation and PCR, and 4 cases by all diagnostic tests. The proportions of positive results by serologic tests, isolation in culture, and PCR using blood specimens were 97% (31/32), 67% (24/36), and 52% (11/21), respectively. The most common *Leptospira* serogroups detected were Hebdomadis in 28 cases (63.6%), followed by Autumnalis in 8 cases (18.2%) (Table 2).

## Discussion

In this population-based study using a mandatory national surveillance program on laboratory-confirmed leptospirosis in children in Okinawa, Japan, we demonstrated that conjunctival suffusion was observed in approximately half of the patients along with other nonspecific systemic symptoms. To our knowledge, this is the first epidemiologic study in English literature on pediatric leptospirosis from Japan, a highly industrialized country.

The symptoms of leptospirosis are nonspecific and range from mild to severe, which contributes to the underdiagnosis of this relatively common disease [3]. According to previous reports of adult patients, common symptoms include abrupt fever, nuchal rigidity, myalgia, and headache in 75–100%; nausea, vomiting, and diarrhea in approximately 50%; and dry coughing in 25–35% of patients. Some patients also had arthralgia, bone pain, throat pain, or abdominal pain [4, 11]. Large-scale clinical reports on pediatric leptospirosis have rarely been published. In our study in children, common clinical features included fever (95%), myalgia (52%), conjunctival suffusion (52%), and gastroenteritis-like symptoms (34%). As compared with a previous report in Okinawa, Japan, which mainly evaluated adult patients, headache, cough, and hemorrhage were less likely to be observed in our pediatric cohort [6] (Table 3). A previous study demonstrated that pediatric patients have a lower risk of death than adult patients during hospitalization for leptospirosis [12]. The reasons for this discrepancy in the presentations between adults and children are unknown.

**Table 3:** Comparison between this study and Tsuha's study, no. (%).

	This study* n = 44	Tsuha’s study† n = 100
Clinical features		
Fever	42 (96)	90 (90)
Myalgia	23 (52)	48 (48)
Conjunctival suffusion	23 (52)	66 (66)
Liver dysfunction	12 (27)	15 (15)
Renal dysfunction	11 (25)	11 (11)
Arthralgia	10 (23)	25 (25)
Jaundice	6 (14)	13 (13)
Headache	6 (14)	76 (76)
Meningitis	4 (9)	2 (2)
Lymphadenopathy	2 (5)	5 (5)
Altered mental status	2 (5)	ND
Rash	1 (2)	0 (0)
Cough	0 (0)	16 (16)
Hemorrhage	0 (0)	5 (5)
Serogroups of Leptospira		
Hebdomadis	28 (64)	25 (34)
Autumnalis	8 (18)	6 (8)
Pyrogenes	3 (7)	14 (19)
Grippotyphosa	2 (5)	1 (1)
Australis	1 (2)	0 (0)
Javanica	0 (0)	5 (7)
Canicola	0 (0)	3 (4)
Not determined	2 (5)**	20 (27)

*This study used Microscopic Agglutination Test.

†Tsuha’s study identified serovars using Korthof media and 74 cases were diagnosed.

**One case: cross reaction, the other case: not tested.

Conjunctival suffusion was observed in approximately half of our patients, which may be an important diagnostic clue. Conjunctival suffusion is one of the most common, unique findings in leptospirosis [13]. This symptom can be observed in other febrile diseases such as typhoid fever, rickettsiosis, Kawasaki disease, and adenovirus infections. However, eye involvement is relatively rare in typhoid fever and rickettsiosis, and these diagnoses can be ruled out by local data on disease endemics and travel history. Kawasaki disease and adenovirus infection commonly manifest as nonspecific febrile conditions with conjunctivitis. However, Kawasaki disease is rare in our patient population (i.e., teenage children) and usually presents with other characteristic findings, including change of lips and oral cavity, polymorphous exanthema, change of peripheral extremities, and acute nonpurulent cervical lymphadenopathy [14]. Adenovirus infection is also rare in our patient population and usually demonstrates upper respiratory and pharyngeal symptoms [15]. Therefore, clinicians should suspect leptospirosis in teenage boys with conjunctival suffusion who reside in or have visited a leptospirosis-endemic area in an industrialized country.

A higher incidence of leptospirosis was observed in children compared with adults, predominantly in boys 10–20 years of age. The rate of leptospirosis in Okinawa, Japan was slightly higher in children than in adults (1.0 and 0.86 per 100,000 population per year, respectively) [9]. Most of our patients were teenage boys. Previously studies reported that the mean age of leptospirosis was 20–40 years of age, and patients <20 years of age and <10 years of age comprised approximately 10% and 1% of all patients, respectively [6, 16–19]. Our data are consistent with these reports. The substantial male preponderance observed in our cohort is also consistent with previous reports [16–18], which is attributed to more frequent involvement in high-risk activities among men compared with women [17]. This hypothesis also applies to our patients, in whom the most common infection route was swimming in a river. This type of recreational activity by children in Japan was assumed to be more commonly performed by teenage boys than girls. Recent studies have also attributed male predominance as part of the explanation for under-diagnosis of the disease in females since females typically present with less severe manifestations associated with lower bacterial loads when compared with males [20, 21].

Suspected acquisition of leptospirosis in our patient cohort was exclusively reported as recreational summer river activity in the northern parts of Okinawa Island and Yaeyama region. Previously reported infection routes of leptospirosis include occupational or recreational activities or travels in a leptospirosis-endemic area, contact with wild animals, and living in unsanitary conditions [3, 22]. However, all reports on suspected infection routes in our patients were recreational river activities (e.g., swimming in a river). This limited acquisition process in our patient cohort can be explained by the lifestyles of children in an industrialized country where involvement in other high-risk activities is substantially less common than in adults or children in resource-limited countries. In addition, unlike the previously reported seasonal pattern from developing countries (i.e., year-round occurrence with a peak in the postsummer season) [16, 17, 23], leptospirosis in our patients exclusively occurred from August to November, which is summer and fall, respectively, in Okinawa. We speculate that this limited seasonal pattern can be attributed to freshwater recreational activities among children on summer vacation and higher precipitation during this season in Okinawa. Leptospirosis outbreaks were reported after a typhoon and a flood in a tropical area [24]. In addition, the two areas in Okinawa where leptospirosis cases were reported are rich in forest and water resources. *Leptospira* spp. is known to thrive in such warm and damp environments [25].

The demographic features and exposure history of pediatric leptospirosis shown in our study offer valuable information for public health and diagnostic purposes. Parents in Okinawa should be notified about the risk of infection by *Leptospira* spp. through river activities in leptospirosis-endemic areas and instructed to seek medical attention in case of development of leptospirosis symptoms. In addition, increased awareness among clinicians is needed to understand that proper history taking plays a crucial role in the diagnosis of leptospirosis. These unique demographic features and exposure history might be shared by different countries with similar economic and climate settings.

Although reliable diagnostic methods for childhood leptospirosis are not well studied, the MAT might be a useful option. Early diagnosis and treatment of leptospirosis are important to prevent complications and alleviate symptoms [26, 27]. Diagnosis of leptospirosis in our surveillance study was based on the National Institute of Infectious Diseases, Japan definitions of confirmed leptospirosis: positive culture, MAT, or PCR results; increase in titer; or histologic findings, which are more strict criteria than the World Health Organization criteria [7, 28]. In our study, approximately 97% (31/32) of serologic tests (i.e., MAT), 67% (24/36) of cultures, and 52% (11/21) of PCR using blood specimens yielded *Leptospira*-positive results. ELISA is often used for epidemiologic investigation because of its high sensitivity and specificity [28]. Our study showed the promising performance of MAT, where all but one patient in our cohort tested by MAT yielded *Leptospira*-positive results. The reported sensitivity of MAT is 30–90% [29–31]. In addition, only two-thirds of patients had positive cultures in our study. The sensitivity of culture for leptospirosis was reported as 5–50% [30, 31], which was influenced by specimen conditions or illness stage [28]. Although only acute-phase specimens were used in our study, there might have been inappropriate choices of culture media or delays in starting cultures. The reported sensitivity of PCR ranges from 50% to 90% [32]. The low positive results by PCR in our patients might be attributed to poor sampling period; blood specimens should be collected at week 1 and urine specimens should be collected at week 2 [25, 32]. However, all tests complement each other for the diagnosis of leptospirosis.

*Leptospira* serogroups Hebdomadis and Autumnalis are commonly observed in children, which might explain the rare occurrence of serious symptoms in children (e.g., hemorrhage). Although the MAT only provides presumptive rather than definitive identification of infecting serogroups, serogroups were identified in 95.5% (42/44) of the cases in our study. Common *Leptospira* serogroups may vary according to geographical location [33]. Compared with Tsuha et al.’s study in Okinawa, which mainly evaluated adult patients [6], Hebdomadis and Autumnalis were more commonly identified in our study (Table 3). This difference is likely attributed to the difference of predominant serogroups in each study period (i.e., 2003–2015 in this study vs. 1974–2015 in Tsuha et al.’s study). Although it is speculation based on limited evidence, adults and children might have different susceptibilities to each *Leptospira* serogroup. However, a direct comparison is difficult because of the limited proportion of cases with identified serogroups in Tsuha et al.’s study. Some serogroups, such as Icterohaemorrhagiae, are known to cause more severe symptoms including alveolar hemorrhage [4, 34, 35]. The absence of these serogroups in our cohort might explain the lack of serious symptoms (e.g., hemorrhage) in our patients. A previous study also reported that pediatric patients have a lower risk of death during hospitalization than adult patients with leptospirosis [12].

There are several limitations associated with this study because of the nature of the surveillance system. First, detailed clinical information such as severity or clinical outcome was not available, which limited in-depth clinical analyses. The reporting form for the surveillance program is designed to request that clinicians mark multiple main signs and symptoms of leptospirosis as either present or absent and offers an area for free descriptions. This ensured reliability of the reported frequency of main findings but might have contributed to underreporting of other minor findings. Second, the potential underdiagnosis and reporting bias on clinical information might have affected the study results. Classification of clinical findings (e.g., hepatic dysfunction, jaundice, meningitis, etc.) was left to the discretion of reporting clinicians. However, Leptospirosis is a notifiable disease in Japan, and this study was conducted by using the information provided from the only central laboratory for leptospirosis in the region. Thus, we included all of the laboratory-confirmed leptospirosis cases in the region.

In conclusion, leptospirosis in children living in Okinawa was reported mainly among teenage boys who had prior recreational exposure (e.g., swimming) in freshwater. Infected children often have nonspecific febrile conditions with myalgia or gastroenteritis-like symptoms. Conjunctival suffusion, however, was observed in approximately half of the patients, which may be an important diagnostic clue given its relatively rare occurrence in other febrile diseases in this population. Cohort studies would be helpful to better understand more detailed clinical manifestations in association with prognosis.

## References

[1] Tullu MS, Karande S. Leptospirosis in children: a review for family physicians. Indian J Med Sci. 2009;63(8):368–78. doi: 10.4103/0019-5359.55893 19770531

[2] Watt G, Padre LP, Tuazon ML, Calubaquib C, Santiago E, Roanoa CP, et al Placebo-controlled trial of intravenous penicillin for severe and late leptospirosis. Lancet. 1988:1(8583):433–5. 2893865 10.1016/s0140-6736(88)91230-5

[3] Bharti AR, Nally JE, Ricaldi JN, Matthias MA, Diaz MM, Lovett MA, et al Leptospirosis: a zoonotic disease of global importance. Lancet Infect Dis. 2003;3(12):757–71. 14652202 10.1016/s1473-3099(03)00830-2

[4] Katz AR, Ansdell VE, Effler PV, Middleton CR, Sasaki DM. Assessment of the clinical presentation and treatment of 353 cases of laboratory-confirmed leptospirosis in Hawaii, 1974–1998. Clin Infect Dis. 2001;33(11):1834–41. doi: 10.1086/324084 11692294 10.1086/324084

[5] Dutta TK, Christopher M. Leptospirosis—an overview. J Assoc Physicians India. 2005; 53:545–51. 16121811

[6] Tsuha S, Taniguchi T, Shiiki S, Narita M, Leung DT. Clinical characteristics of laboratory-confirmed leptospirosis in Okinawa, Japan, 1974–2015: high incidence of Jarisch-Herxheimer reaction. Trans R Soc Trop Med Hyg. 2016;110(9): 558–65. doi: 10.1093/trstmh/trw061 27744340 10.1093/trstmh/trw061

[7] National Institute of Infectious Disease, Japan. Leptospirosis manual [In Japanese]. [cited 2017 May 27]. https://www0.niid.go.jp/niid/reference/leptospirosis-manual.pdf

[8] Okinawa Prefectural Government, Department of Public Health and Medical Care, Institute of Health and Environment. Trends of Leptospirosis in Okinawa Prefecture. [cited 2017 May 27]. http://www.pref.okinawa.jp/site/hoken/eiken/kikaku/kansenjouhou/documents/leptospirosis_2015.pdf

[9] Okinawa Prefectural Government, Department of Planning, Statistics Division. Population Estimate. [cited 2017 May 27]. http://www.pref.okinawa.jp/toukeika/estimates/estimates_suikei.html

[10] Ministry of Education, Culture, Sports, Science and Technology and Ministry of Health, Labour and Welfare. Ethical Guidelines for Medical and Health Research Involving Human Subjects. [cited 2017 May 27]. http://www.mhlw.go.jp/file/06-Seisakujouhou-10600000-Daijinkanboukouseikagakuka/0000080278.pdf

[11] Bertherat E, Renaut A, Nabias R, Dubreuil G, Georges-Courbot MC. Leptospirosis and Ebola virus infection in five gold-panning villages in northeastern Gabon. Am J Trop Med Hyg. 1999;60(4):610–5. 10348236 10.4269/ajtmh.1999.60.610

[12] Lopes AA, Costa E, Costa YA, Sacramento E, de Oliveira Junior AR, Lopes MB, et al Comparative study of the in-hospital case-fatality rate of leptospirosis between pediatric and adult patients of different age groups. Rev Inst Med Trop Sao Paulo. 2004;46(1):19–24. 15057329 10.1590/s0036-46652004000100004

[13] Lin CY, Chiu NC, Lee CM. Leptospirosis after typhoon. Am J Trop Med Hyg. 2012;86(2):187–8. doi: 10.4269/ajtmh.2012.11-0518 22302844 10.4269/ajtmh.2012.11-0518 PMC3269263

[14] Ayusawa M, Sonobe T, Uemura S, Ogawa S, Nakamura Y, Kiyosawa N, et al Revision of diagnostic guideline for Kawasaki disease (the 5th revised edition). Pediatr Int. 2005;47(2):232–4. doi: 10.1111/j.1442-200x.2005.02033.x 15771703 10.1111/j.1442-200x.2005.02033.x

[15] Dominguez O, Rojo P, de Las Heras S, Folgueria D, Contreras JR. Clinical presentation and characteristics of pharyngeal adenovirus infections. Pediatr Infect Dis J. 2005;24(8):733–4. 16094232 10.1097/01.inf.0000172942.96436.2d

[16] Jansen A, Schoneberg I, Frank C, Alpers K, Schneider T, Stark K. Leptospirosis in Germany, 1962–2003. Emerg Infect Dis. 2005;11(7): 1048–54. doi: 10.3201/eid1107.041172 16022779 10.3201/eid1107.041172 PMC3371786

[17] Katz AR, Buchholz AE, Hinson K, Park SY, Effler PV. Leptospirosis in Hawaii, USA, 1999–2008. Emerg Infect Dis. 2011;17(2):221–6. doi: 10.3201/eid1702.101109 21291592 10.3201/eid1702.101109 PMC3204774

[18] Goris MG, Boer KR, Duarte TA, Kliffen SJ, Hartskeerl RA. Human leptospirosis trends, the Netherlands, 1925–2008. Emerg Infect Dis. 2013;19(3):371–8. doi: 10.3201/eid1903.111260 23622144 10.3201/eid1903.111260 PMC3647640

[19] Traxler RM, Callinan LS, Holman RC, Steiner C, Guerra MA. Leptospirosis-associated hospitalizations, United States, 1998–2009. Emerg Infect Dis. 2014;20(8):1273–9. doi: 10.3201/eid2008.130450 25076111 10.3201/eid2008.130450 PMC4111189

[20] Jansen A, Stark K, Schneider T, Schoneberg I. Sex differences in clinical leptospirosis in Germany: 1997–2005. Clin Infect Dis. 2007; 4:e69–72. 10.1086/513431 17407027

[21] Agampodi SB, Matthias MA, Moreno AC, Vinetz JM. Utility of quantitative polymerase chain reaction in leptospirosis diagnosis: associateon of level of leptospiremia and clinical manifestations in SriLanka. Clin Infect Dis. 2012; 54: 1249–55. doi: 10.1093/cid/cis035 22354922 10.1093/cid/cis035 PMC3404689

[22] Victoriano AF, Smythe LD, Gloriani-Barzaga N, Cavinta LL, Kasai T, Limpakarnjanarat K, et al Leptospirosis in the Asia Pacific region. BMC Infect Dis. 2009; 9:147 doi: 10.1186/1471-2334-9-147 19732423 10.1186/1471-2334-9-147 PMC2749047

[23] van Alphen LB, Ethelberg S, Villumsen S, Krogfelt KA. Trends in human leptospirosis in Denmark, 1980 to 2012. Euro Surveill. 2015;20(9). 25764192

[24] Su HP, Chan TC, Chang CC. Typhoon-related leptospirosis and melioidosis, Taiwan, 2009. Emerg Infect Dis. 2011;17(7):1322–4. doi: 10.3201/eid1707.101050 21762606 10.3201/eid1707.101050 PMC3381404

[25] Levett PN. Leptospirosis. Clin Microbiol Rev. 2001;14(2):296–326. doi: 10.1128/CMR.14.2.296-326.2001 11292640 10.1128/CMR.14.2.296-326.2001 PMC88975

[26] McClain JB, Ballou WR, Harrison SM, Steinweg DL. Doxycycline therapy for leptospirosis. Ann Intern Med. 1994;100(5):696–8. 10.7326/0003-4819-100-5-696 6712032

[27] Watt G, Padre LP, Tuazon ML, Calubaquib C, Santiago E, Ranoa CP, et al Placebo-controlled trial of intravenous penicillin for severe and late leptospirosis. Lancet. 1998;1(8583):433–5. 10.1016/s0140-6736(88)91230-5 2893865

[28] World Health Organisation and International Leptospirosis Society. Human Leptospirosis: Guidance for Diagnosis, Surveillance and Control. [cited 2017 May 27]. http://www.who.int/csr/don/en/WHO_CDS_CSR_EPH_2002.23.pdf

[29] Cumberland P, Everard CO, Levett PN. Assessment of the efficacy of an IgM-elisa and microscopic agglutination test (MAT) in the diagnosis of acute leptospirosis. Am J Trop Med Hyg. 1999;61(5):731–4. 10586903 10.4269/ajtmh.1999.61.731

[30] Limmathurotsakul D, Turner EL, Wuthiekanun V, Thaipadungpanit J, Suputtamongkol Y, Chierakul W, et al Fool’s gold: Why imperfect reference tests are undermining the evaluation of novel diagnostics: a reevaluation of 5 diagnostic tests for leptospirosis. Clin Infect Dis. 2012;55(3):322–31. doi: 10.1093/cid/cis403 22523263 10.1093/cid/cis403 PMC3393707

[31] Hartskeerl RA, Collares-Pereira M, Ellis WA. Emergence, control and re-emerging leptospirosis: dynamics of infection in the changing world. Clin Microbiol Infect. 2011;17(4):494–501. doi: 10.1111/j.1469-0691.2011.03474.x 21414083 10.1111/j.1469-0691.2011.03474.x

[32] de Abreu Fonseca C, Teixeira de Feitas VL, Calo Romero E, Spinosa C, Arroyo Sanches MC, de Silva MV, et al Polymerase chain reaction in comparison with serological tests for early diagnosis of human leptospirosis. Trop Med Int Health. 2006;11(11):1699–707. doi: 10.1111/j.1365-3156.2006.01727.x 17054750 10.1111/j.1365-3156.2006.01727.x

[33] Bolin CA. Diagnosis of leptospirosis: a reemerging disease of companion animals. Semin Vet Med Surg (Small Anim). 1996;11(3):166–71. 8942213 10.1016/s1096-2867(96)80029-6

[34] Yersin C, Bovet P, Merien F, Wong T, Panowsky J, Perolat P. Human leptospirosis in the Seychelles (Indian Ocean): a population-based study. Am J Trop Med Hyg. 1998;59(6):933–40. 9886203 10.4269/ajtmh.1998.59.933

[35] Guerrier G, Hie P, Gourinat AC, Huguon E, Polfrit Y, Goarant C, et al Association between age and severity to leptospirosis in children. PLoS Negl Trop Dis. 2013;7(9):e2436 doi: 10.1371/journal.pntd.0002436 24086780 10.1371/journal.pntd.0002436 PMC3784464

